# Field-emission from quantum-dot-in-perovskite solids

**DOI:** 10.1038/ncomms14757

**Published:** 2017-03-24

**Authors:** F. Pelayo García de Arquer, Xiwen Gong, Randy P. Sabatini, Min Liu, Gi-Hwan Kim, Brandon R. Sutherland, Oleksandr Voznyy, Jixian Xu, Yuangjie Pang, Sjoerd Hoogland, David Sinton, Edward Sargent

**Affiliations:** 1 Department of Electrical and Computer Engineering, University of Toronto, 35 St George Street, Toronto, Ontario, Canada M5S 1A4; 2 Department of Mechanical and Industrial Engineering, University of Toronto, 5 King's College Road, Toronto, Ontario, Canada M5S 3G8

## Abstract

Quantum dot and well architectures are attractive for infrared optoelectronics, and have led to the realization of compelling light sensors. However, they require well-defined passivated interfaces and rapid charge transport, and this has restricted their efficient implementation to costly vacuum-epitaxially grown semiconductors. Here we report solution-processed, sensitive infrared field-emission photodetectors. Using quantum-dots-in-perovskite, we demonstrate the extraction of photocarriers via field emission, followed by the recirculation of photogenerated carriers. We use *in operando* ultrafast transient spectroscopy to sense bias-dependent photoemission and recapture in field-emission devices. The resultant photodiodes exploit the superior electronic transport properties of organometal halide perovskites, the quantum-size-tuned absorption of the colloidal quantum dots and their matched interface. These field-emission quantum-dot-in-perovskite photodiodes extend the perovskite response into the short-wavelength infrared and achieve measured specific detectivities that exceed 10^12^ Jones. The results pave the way towards novel functional photonic devices with applications in photovoltaics and light emission.

## Abstract (web summary)

Efficient implementation of quantum dot and well architectures are restricted to costly vacuum-epitaxially-grown semiconductors. The authors use quantum dots in perovskite to build field-emission photodiodes that are sensitive across the visible and into the short-wavelength infrared.

The interaction of photons and electrons at the nanoscale enables optoelectronic devices such as solar cells, light-emitting diodes and photodetectors. Photodetectors have traditionally been made using single-crystalline materials such as silicon and III–V semiconductors. Of interest for many applications such as medical imaging, machine vision, surveillance and communications is the infrared region of the spectrum. Quantum dot and quantum well architectures, which exploit the confinement of charges at the nanoscale, are particularly attractive to address this region. They benefit from tunable confined volumes that emit carriers into a host phase. For this process to be efficient, well-defined interfaces and high mobilities in the transport phase are required. These demanding requirements have restricted thus far the implementation of quantum heterostructures to the domain of high-temperature epitaxy.

The emergence of new nanostructured materials that benefit from low-temperature solution processing holds promise for the scalable manufacturing of large-area devices. These materials also offer an attractive toolkit of device-enabling electronic materials properties programmed at the nanoscale, including controlled doping^1^,^2^ and energy level tuning^3^,^4^. The performance of solution-processed photodetectors, and in particular those based on organic semiconductors and colloidal quantum dots (CQDs), has rapidly increased and become competitive with that of silicon and III–V semiconductors^5^,^6^,^7^,^8^.

Early CQD photodetectors were based on the sensitization of host transport polymer matrices that served as a channel for carrier collection and transport^9^,^10^,^11^. These proof-of-concept devices were limited by the morphology of composites and the electronic properties of host materials. The low mobilities of organic materials were particularly limiting, holding performance well below that of heteroepitaxial quantum dot and quantum well photodetectors.

In principle, a well-engineered heterodevice can benefit from suppressed thermal generation—managed through carrier confinement and band engineering—and the separation of sensitization and transport, the latter enabled by the use of crystalline and low-defect materials^12^. Until recently, implementing lattice-matched heterocomposite devices for infrared photodetection has remained a challenge in solution-processed soft materials, since several requirements need to be fulfilled simultaneously: an electronically superior host-transport material with a low density of defects and high mobility, the presence of a tunable sensitizer, and a materials-processing strategy that enables the compatible incorporation of the sensitizer into the host. Ideally, the interface between the host material and sensitizer should be epitaxial to ensure a low density of localized defects and to minimize recombination channels. All this must be achieved with a device architecture that favours the injection of photocarriers into, and collection of photogenerated charges from within, the transport phase. Fulfilling this requirement ensures high quantum efficiency (high primary photocurrent) as well as gain (high secondary photocurrent).

In view of their attractive optoelectronic properties, organometal halide perovskite semiconductors have recently been explored for photodetection applications. Perovskite photodiodes have been reported with large-specific detectivity^13^, fast temporal response^14^, high dynamic range^15^ and colour selectivity^16^,^17^,^18^. This performance has been enabled by a set of compelling electronic properties that include high and balanced carrier mobilities^19^, low-trap densities^20^ and high-absorption coefficients^21^,^22^. However, in spite of these advantageous characteristics, the bandgap of organo-lead perovskites (A)PbI_3_ (A=MA, FA, Cs^+^) precludes their application in the important domain of short-wavelength infrared (SWIR) photodetection^23^,^24^,^25^,^26^.

The bandgap of CQDs can, in contrast with those of the best perovskites, be tuned into the SWIR^27^. However, the processing steps required for the assembly of CQDs into films are typically associated with the formation of an appreciable density of electronic trap states arising from incompletely-passivated nanoparticle surfaces^28^. A new type of semiconducting material, one that consists of CQDs embedded in an epitaxially grown perovskite matrix, has been recently reported^29^. These quantum-dot-in-perovskite (QDiP) materials combine the advantages of each: CQD sensitization (therefore, bandgap tunability), superior passivation of the CQD surface states in view of their epitaxially matched interface, and the excellent carrier transport properties with low-trap density and high mobility of organohalide perovskites. Therefore, in principle, QDiP solids offer an attractive platform for infrared photodetection.

In this work we report the implementation of field-emission solution-processed devices. We take advantage of the benefits of QDiP materials to build photodiodes that are sensitive across the visible and into the SWIR. Their experimental specific detectivities (D*∼4 × 10^12^ Jones) in the SWIR exceed by over two-fold those of the best-performing CQD photodiodes in this spectral region. Their excellent performance arises from the synergy between CQD sensitization, and a dedicated charge transport channel. The approach accomplishes the harvest and extraction of charges photogenerated in the CQDs *via* field emission into the perovskite phase. Our design strategy, discussed in detail herein, optimizes carrier ejection into the perovskite and transport through this host medium, and minimizes recapture to the CQDs. The resulting field-emission QDiP photodetectors exhibit a bandwidth of 60 kHz, an above-unity gain, noise current densities of 0.1 pA Hz^−1/2^ and a linear dynamic range exceeding 60 dB.

## Results

### Field-emission QDiP photodiodes

**Figure 1:**
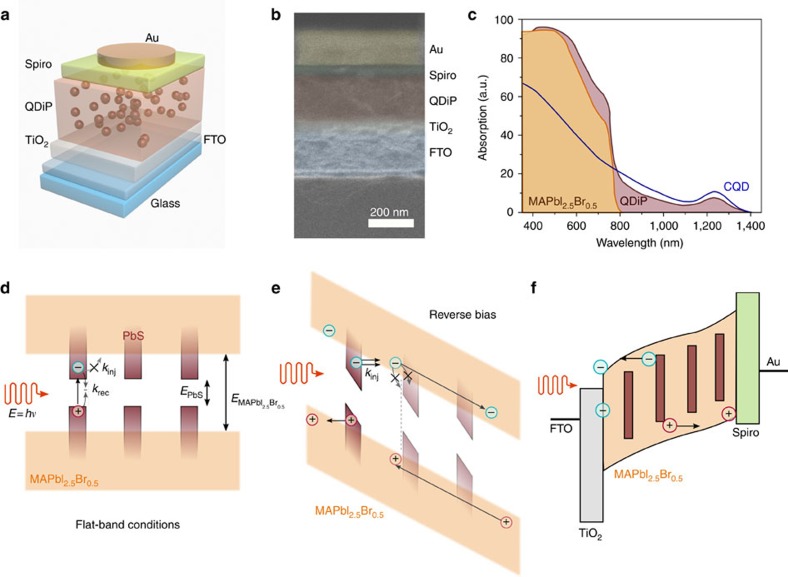
Field-emission QDiP photodetector. (**a**) Photodiode device schematic. The QDiP photoactive layer is sandwiched between TiO_2_ (electron-transport layer) and spiro-MeOTAD (hole-transport layer). (**b**) Cross-sectional SEM of a typical device, color-coded overlay for clarity. (**c**) Absorption of pure MAPbI_2.5_Br_0.5_ perovskite, QDiP (1:1 ratio) and colloidal quantum dot (1 eV) films. Above 800 nm MAPbI_2.5_Br_0.5_ is not photosensitive, whereas the QDiP extends up to 1,400 nm. (**d**) For a type-I heterojunction, at flat band conditions photogenerated charges in the CQD phase cannot escape and lose their energy via recombination. Charges in the perovskite will eventually get trapped and recombine through the quantum dots. (**e**) Under sufficient reverse bias, carriers can tunnel into the MAPbI_2.5_Br_0.5_ host assisted by the high electric field and be collected. The transport matrix needs therefore to combine high mobilities with minimized recombination at the dot's interface in order to repeal recapture probability. (**f**) Proposed operation of the field-emission QDiP photodiode.

The device architecture (Fig. 1a) comprises a TiO_2_ electron-transport layer (50 nm), deposited using atomic layer deposition, on top of a transparent conductive fluorine-doped tin oxide (FTO) electrode. A 250 nm thick QDiP film is formed on top. The host perovskite matrix consists of MAPbI_2.5_Br_0.5_, where the iodide and bromide ratio has been optimized to achieve better lattice matching with the embedded dots and thus both passivation and excellent transport characteristics^30^. The quantum dots were synthesized as reported elsewhere^31^ and the size of the CQDs tuned to yield an exciton peak at 1,240 nm (∼1 eV). This is followed by a 50 nm layer of Spiro-MeOTAD and 200 nm of gold to form the hole-extraction layer. A cross-sectional scanning electron micrograph (SEM) of typical devices reveals the thicknesses and highlights the uniform character of each layer (Fig. 1b).

The absorption spectra of individual MAPbI_2.5_Br_0.5_, CQD and QDiP films of similar thicknesses reveal the contributions from each phase (Fig. 1c). MAPbI_2.5_Br_0.5_ shows a strong absorption throughout the visible with a cutoff wavelength of 800 nm (ref. 30). The absorption of the CQD film begins at 1,400 nm (∼0.88 eV), shows a first exciton peak at 1,240 nm (∼1 eV), and increases for shorter wavelengths. The absorption of the QDiP film matches well with that of the CQD film in the infrared. The invariance of the position of the exciton peak from solution to film formation indicates evenly distributed (non-aggregated), monodispersed quantum dots dispersed in the host perovskite matrix^30^. At 800 nm the absorption increases, consistent with that of the perovskite matrix.

In the absence of external electric fields, photogenerated excitons are expected to be confined inside CQDs due to the type-I heterojunction formed between the CQD (∼1 eV) and the host MAPbI_2.5_Br_0.5_ (∼1.6 eV; Fig. 1d), and recombined either through radiative or non-radiative channels. This should change under the presence of a suitably large electric field (Fig. 1e): excitons photogenerated in the quantum dots will then separate, and the charges may be ejected from the dots, under the action of the field, into the host perovskite matrix. The appropriate point of operation needs to ensure efficient photocarrier escape, and circulation that is rapid enough to avoid excessive recapture. Emitted electrons or holes can then be extracted and reinjected at the electrodes, resulting in a multiplicative (that is, one exhibiting gain) photocurrent (Fig. 1f).

### Device operation and Fowler–Nordheim tunnelling

**Figure 2:**
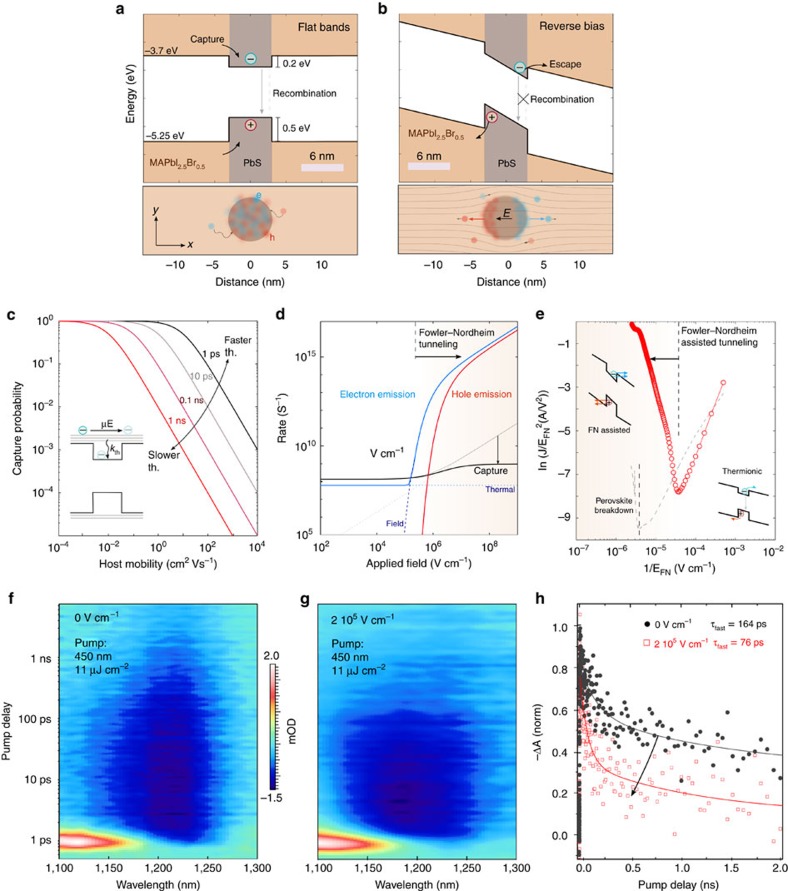
Exciton harnessing and FN emission. Band alignment of QDiP solids as calculated with a SCAPS model at (**a**) no bias and (**b**) reverse bias conditions. In the absence of an imposed electric field photogenerated charges are uniformly distributed across the dot (bottom panels). At sufficient reverse bias conditions they are spacially separated and can inject into the MAPbI_2.5_Br_0.5_ host, favouring carrier recirculation over capturing. (**c**) Carrier capture probability for different thermalization rates as a function of host mobility. High mobilities are required in order for the carriers not to be re-captured (**d**) electron/hole emission and capture rates as a function of applied field for the QDiP system. Above 0.1 MV cm^−1^ FN emission overcomes carrier capture. (**e**) FN diagram of a QDiP device reveals the region where field-emission tunelling is the dominant injection mechanism. *In situ* ultrafast transient absorption maps of QDiP devices at (**f**) 0 MV cm^−1^ and (**g**) 0.2 MV cm^−1^ reveals carrier injection within 100 ps. (**h**) The dynamics of the exciton bleach peak at 1,240 nm follow a biexponential behaviour with a fast lifetime accelerated from 164±32 ps to 76±9 ps (Supplementary Fig. 2). A fs-pump wavelength of 450 nm with a fluence of 11 μJ cm^−2^ was used in each configuration.

We sought first to evaluate the conditions and means under which photoexcited charges in the quantum dots could be ejected into, and collected from within, the MAPbI_2.5_Br_0.5_ matrix. We carried out numerical simulations of photoexcitation, escape and recapture processes, as well as transport, in the QDiP solid (Fig. 2a). The energy landscape of a simplified one-dimensional linear stack of perovskite-dot-perovskite clarifies the different injection regimes. For a standard CQD with an exciton peak of 1 eV, band offsets of 0.5 and 0.2 eV with the top and bottom of the perovskite valence and conduction bands, respectively, ensure that photogenerated electron and hole densities reside within the CQDs (bottom panel). Carriers in the perovskite phase are captured in the quantum dots and undergo recombination.

Under reverse bias conditions (Fig. 2b), the applied electric field is distributed across the QDiP solid, with a higher intensity in the PbS quantum dots in light of their lower dielectric permittivity (*ɛ*_PbS_ ∼22 and 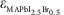
∼70)^32^,^33^. The larger electric field in the quantum dots promotes the separation of the electron and hole wavefunctions (bottom panel) facilitating ejection, via tunnelling or emission over the barrier, into the perovskite phase. The simulated photocurrent for this material configuration (Supplementary Fig. 1) monotonically increases under increasing electric field, and then begins to saturate above 0.2 MV cm^−1^, where the extraction efficiency reaches a maximum.

After carriers escape into the perovskite, they could be potentially re-captured into the dots, as determined by the competition between carrier thermalization and extraction time (Fig. 2c). A given cooling rate (*k*_th_) will result in a mobility (*μ*_host_) threshold for a given applied electric field (*E*_N_). Assuming an external field of 1 MV cm^−1^, a typical *k*_th_ in the range of 1–10 ps would require *μ*_host_ on the order of 10 cm^2^·(V s)^−1^ to reduce the capture probability below 0.1. This underpins the necessity of an excellent transport material for this architecture.

Different types of tunnelling mechanisms can provide channels for charge injection from the photoexcited dots into the surrounding perovskite matrix. Schottky and thermionic emission are expected to dominate for low and moderate electric fields, and to become outpaced by field assisted (Fowler–Nordheim, (FN)) at higher biases^34^. Therefore, the rate at which confined carriers can escape from the quantum dots, as well as the capture rate, depend strongly on the applied electric field (Fig. 2d). Below 0.1 MV cm^−1^, thermionic emission dominates. Above this threshold, FN tunnelling increases until it becomes the dominant mechanism for electron escape. The capture rate follows a similar trend but requires above 1 MV cm^−1^ in view of fast transport throughout the QDiP solid. As a result, the majority of the photogenerated carriers will be collected once the field exceeds 0.1 MV cm^−1^.

To assess experimentally the dominant tunnelling mechanism in the QDiP solid, we measured the current density (*J*) through a device as a function of the applied reverse electric field. The FN plot (Fig. 2e) provides strong evidence that field-assisted FN tunnelling is the dominant mechanism for charge injection at electric fields >3 × 10^4^ V·cm^−1^, which corresponds to reverse biases around ∼1 V. This agrees well with the analytical and simulated predicted values^35^. We note that this range of voltages is well below the breakdown voltage of the perovskite matrix, above ∼7 V (Fig. 2e, Supplementary Note 1).

We then monitored the ultrafast dynamics of the photogenerated carriers using transient absorption spectroscopy (Fig. 2f–h). The litmus test of efficient net photocarrier emission is as follows: fast absorption kinetics in the quantum dot phase, accompanied by a slower decay in the perovskite, will indicate net emission from dots into perovskite. Further, a bias-dependence to these trends will signal a role of field. At 0 V cm^−1^ the ground state bleach signal of the quantum dots decays bi-exponentially with a fast lifetime component of 164 ps and a slower component of 1.5 ns (Supplementary Note 2 and Supplementary Fig. 2). With the application of an electric field of 2 × 10^5^ V cm^−1^, the quantum dot bleach shows accelerated decay dynamics (*τ*∼76 ps) under the same photoexcitation conditions. This significantly accelerated decay stems from the new carrier extraction pathway introduced by the applied field: photogenerated excitons are dissociated into charge carriers, and then emitted into the perovskite matrix, avoiding recapture.

We observe the opposite trend while probing the perovskite phase: the decay rate decelerates upon the application of the electric field, indicating retention of carriers in the perovskite phase (Supplementary Fig. 3 and Supplementary Table 2).

In contrast, the photocarrier dynamics of pure perovskite and quantum dot devices show no appreciable dependence on bias (Supplementary Figs 4 and 5).

### Optoelectronic characterization

After we identified operation guidelines of this material platform, we then sought to characterize the optoelectronic performance of our QDiP photodiodes. The current–voltage characteristics under dark conditions reveal the rectifying behaviour of the devices. Further, the QDiP devices show lower dark currents than do purely CQD-based diodes (Supplementary Fig. 6).

**Figure 3:**
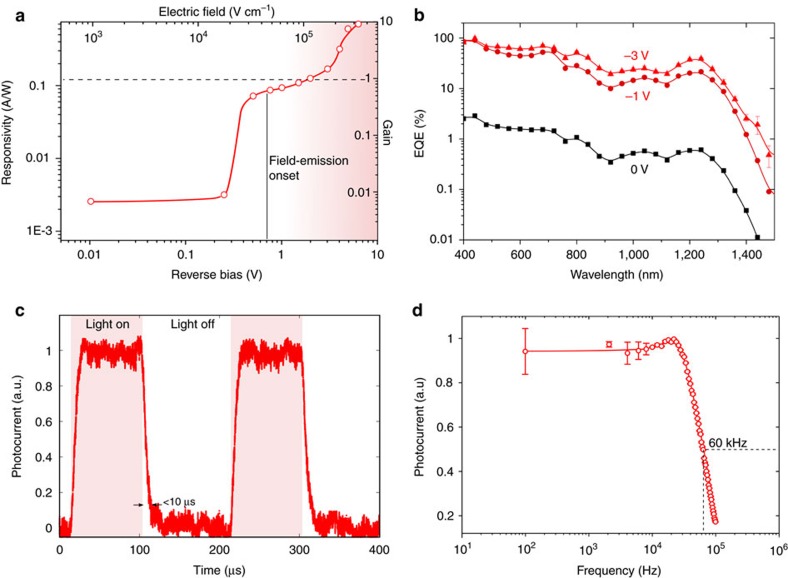
Photodetector performance. (**a**) The responsivity (left axis) as a function of reverse bias under 975 nm (1.31 eV) illumination displays a sharp increase at the field-emission onset, and a photomultiplicative gain (right axis) exceeding unity above 2 V reverse bias. An increase of over a two-orders of magnitude is recorded before breakdown occurs. (**b**) The EQE spectra at different biases showcases the contribution of the quantum-dots at the infrared. A 60-fold enhancement is obtained at the exciton peak (Supplementary Fig. 8). (**c**) The photoresponse dynamics at 1 V reverse bias are characterized by a sub 10 μs fall time with a corresponding 3-dB bandwidth of 60 kHz. (**d**) Responsivity-bias and time response were characterized using 975 nm monochromatic illumination at a fluence of 100 μW cm^−2^. Error bars correspond to the s.d. over 20 measurements.

We first evaluated the bias-dependence of the photoresponse and measured the responsivity (*R*) and estimated gain (*G*) for a wide range of biases under 975 nm illumination, where the QDs are the only absorbing phase (Fig. 3a). The photoresponse remains constant (∼2.2 mA W^−1^) up to 0.3 V reverse bias. A 45-fold increase in responsivity is obtained at 0.5 V reverse bias, as photogenerated charges in the QDs start to be emitted into the perovskite matrix. Beyond −2 V, the unity gain threshold is exceeded for this wavelength. We ascribe this photomultiplicative process to the imbalance of electron and hole injection rates (Supplementary Fig. 7). A maximum gain of 10 is achieved at 6 V reverse bias (Supplementary Note 3, Supplementary Fig. 8).

The spectral response of the detector at short-circuit conditions extends to 1,400 nm, confirming the photoactive contribution of the QD phase in the SWIR (Fig. 3b). The external quantum efficiency (EQE) is in this case low, as most of the photogenerated carriers meet and eventually recombine in the CQDs (Fig. 2). A maximum EQE of 3 and 0.6% are observed at 400 nm and at the 1,240 nm exciton peak, respectively.

As the reverse bias is increased to −1 V, this picture changes dramatically, and a significant improvement in the EQE occurs, reaching 20% at the exciton peak. When we further increase the applied bias to −3 V, the EQE saturates in the visible region (∼90% at 400 nm) and increases to 40% at 1,240 nm. The emission efficiency increase with bias is more prominent at long wavelengths, and a 60-fold improvement is observed at −3 V reverse voltage (Supplementary Note 4, Supplementary Figs 9 and 10).

The temporal response of the device was characterized in the fluence regime of linear operation. We observe rise and fall times below 10 μs and a corresponding 3 dB bandwidth of 60 kHz (Fig. 3c,d). A maximum gain × bandwidth product of 180 kHz was measured at 3 V reverse bias (Supplementary Fig. 11).

### Field-emission in QDiP enables record sensitivity

**Figure 4:**
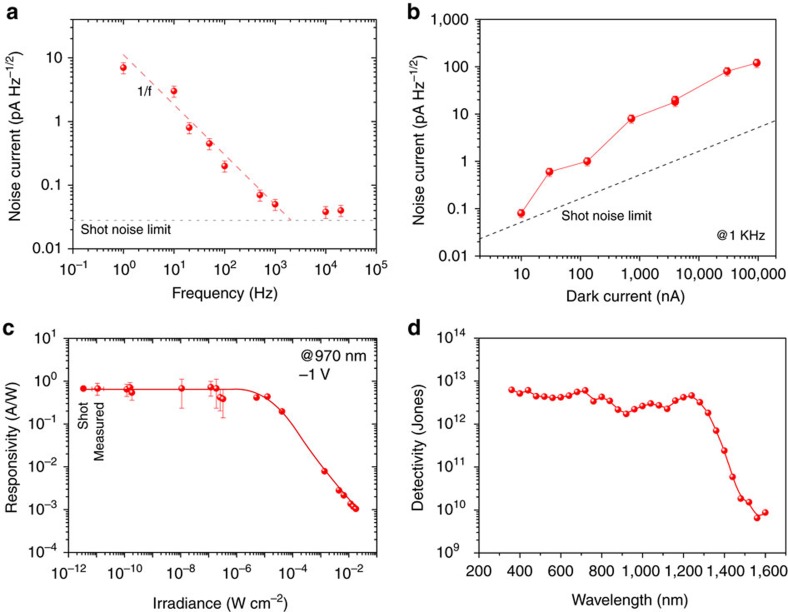
Noise and sensitivity. (**a**) The noise current dependence with frequency at 0 V reveals a 1/*f* behaviour that approaches the shot-noise limit for frequencies greater than 1 kHz. (**b**) At 1 kHz frequency, the measured noise is dominated by its shot component, with an increasing contribution of G–R at higher dark currents. (**c**) Responsivity and noise equivalent power (NEP) at 1 V reverse bias and 275 K. A dynamic range of ∼60 dB is obtained. (**d**) Specific detectivity spectrum at the optimum operation conditions. Extended SWIR sensitivity with a detectivity of 4 × 10^12^ Jones at the CQD exciton peak is obtained. Error bars correspond to the s.d. over 20 measurements.

We then proceeded to evaluate the sensitivity of the QDiP photodiodes and characterized their noise under various operating frequency and bias conditions (Fig. 4a,b). At 1 Hz modulation and short-circuit conditions, the measured noise spectral density (∼30 pA Hz^−1/2^) exceeds the one predicted at the shot-noise limit (*i*_SN_=(2*qI*_d_)^1/2^=0.04 pA Hz^−1/2^; Fig. 4a) by more than two orders of magnitude. This is gradually reduced as the operating frequency increases and flattens around 1 kHz to a value 50% above the shot-noise limit. This characteristic is typical of flicker (1/*f*) noise^36^ and is attributed to bulk defects in the perovskite matrix^37^.

Under increasing reverse bias conditions, and a fixed frequency of 1 kHz, the noise current increases and gradually deviates from the shot-noise limit (Fig. 4b). We associate this phenomenon to the increasing contribution of generation–recombination (G–R) noise, which is directly proportional to the photomultiplicative gain^38^,^39^. Lowering the device operation temperature is expected to result in improved sensitivity, as the noise contribution is reduced from its thermal, shot and G–R components, which we confirmed experimentally (Supplementary Note 5, Supplementary Figs 12–14). Simply by reducing the operation temperature by 25 K, we were able to obtain an order of magnitude reduction in the noise current spectral density to below 0.3 pA Hz^−1/2^.

The responsivity (*R*) at a number of illumination intensities under 975 nm excitation shows a power-independent behaviour (that is, a linear relation between photocurrent and power) across 6 decades up to 0.1 mW cm^−2^. This yields a linear dynamic range of 60 dB (Fig. 4c). The diminution in responsivity at higher irradiances suggests the presence of nonlinear photomultiplication mechanisms, where the increasing occupation of trap states results in a reduction of trap-assisted gain processes^40^.

The measured specific detectivity (D*=*R* × *A*^1/2^/*i*_*n*_) reaches 5 × 10^12^ Jones through the visible and 4 × 10^12^ Jones at the exciton peak (1,240 nm; Fig. 4d). This represents more than a doubling compared to the best previously-reported CQD SWIR photodiodes^41^. The performance of other commercial significant infrared photodetectors is shown for comparison (Supplementary Fig. 15, Supplementary Table 3). QDiP devices were stable under the stringent (1 V) operation condition under continuous operation over an initial 100 h (Supplementary Note 6, Supplementary Figs 16 and 17).

## Discussion

This work demonstrated efficient field-emission solution-processed infrared photodetectors. We achieved this by implementing a QDiP into a photodiode architecture and programming its operation in the field-emission regime. This exploited both collection of photogenerated carriers and minimized noise and led to superior optoelectronic performance that extended the photoresponse of perovskite photodiodes well into the SWIR. The resulting devices showcase specific detectivities on par with Si and organohalide perovskite photodiodes in the visible regime, and surpass that of the best CQD photodiodes in the SWIR regime, where we achieved D*∼4 × 10^12^ Jones.

The all-solution-processed approach could enable sensitive multispectral imaging in applications that demand efficient SWIR sensing such as night vision, surveillance, machine vision and gesture recognition. Further improvements and device function can be envisaged by modifying the host perovskite matrix: the use of wider bandgap perovskites could open the door for-IR sensitive, visible-blind photodetection.

The reported field-emission solution-processed optoelectronic architecture exploits a set of physical effects previously attained only through costly heteroepitaxy. Potential energy harvesting applications include cascade intermediate-band photovoltaics capable of collecting the infrared solar spectrum with high open-circuit voltages^42^, and photon upconverters for light-emission^43^.

## Methods

### Material synthesis and device fabrication

TiO_2_ electron-transport layers (50 nm) were deposited on patterned F-doped SnO_2_ (FTO, Pilkington, TEC 15) substrates. This was followed by drying at 130 °C for 10 min and calcination at 500 °C for 1 h. The TiO_2_ precursor solution was prepared by dissolving 890 mg titanium isopropoxide and 30 mg HCl (37%) solution in 8 ml ethanol, and the mixture was stirred at room temperature overnight before use. The resulting TiO_2_ films were immersed in 120 mM of TiCl_4_ aqueous solution at 70 °C for 1 h and then heated at 500 °C for another 30 min. QDiP solid thin film were synthesized and fabricated as reported previously^30^. Briefly, the desired amount of perovskite precursor (PbI_2_ and PbBr_2_ with one quarter weight ratio of CH_3_NH_3_I) in butylamine was added to the solution exchanged PbS CQDs. The mass ratio of the CQDs to perovskite in solution for the optimized devices is 1:1 (quantum dots to perovskite PbX_2_ precursor ratio). This corresponds to a mass ratio of approximately 1:1.33 in the final QDiP film (quantum dots to perovskite w/w). The resulting colloid was then spin-coated (6,000 r.p.m., 10 s) onto the substrates, and then annealed at 70 °C for 10 min in an N_2_ atmosphere to remove excess butylamine. For perovskite growth, methylammonium halide mixed solution (10 mg ml^−1^ in isopropanol) was drop-cast on the film and removed after 30 s by spin coating (6,000 r.p.m., 10 s), after which the film was soaked in pure isopropanol for 10 s, and the substrate was again spun (6,000 r.p.m., 10 s) for complete removal of the residual solvent. QDiP films were annealed again at 70 °C for 10 min in an N_2_ glovebox. The hole-transport layer was spin-coated onto the resulting films at 4,000 r.p.m. for 30 s using a chlorobenzene solution containing 63 mg ml^−1^ of spiro-OMeTAD, 20 μl of tert-butylpyridine and 70 μl of bis(trifluoromethane)sulfonimide lithium salt (170 mg ml^−1^ in acetonitrile). Top gold electrodes (100 nm) were deposited using an Angstrom Engineering deposition system in an Innovative Technology glovebox through electron-beam deposition at a rate of 0.4 Å s^−1^. A shadow mask was used to define the electrical active area of the devices (*A*_d_∼0.07 cm^2^). The fabrication of these devices is in compliance with RoHs regulations (Supplementary Note 7).

### Material characterization

SEM imaging: cross-sectional SEMs of typical devices were acquired using a Hitachi SU-8230 apparatus after cleaving the samples with a diamond scriber. Absorption spectroscopy was performed with a Perkin Elmer Lambda 900 spectrometer with an integrating sphere attachment. Total absorption was calculated from transmission and reflection as *A*=1−*R*−*T*.

### Optoelectronic characterization

Current–voltage characteristics of the photodiodes were recorded using a Keithley 2400 source metre. Spectral responsivity was measured by illuminating the device through a 0.049 cm^2^ aperture with a collimated 220 Hz chopped light source (450 W xenon lamp through a monochromator with order-sorting filters). The power was measured with calibrated Newport 818-UV and infrared power metres, and is lies in the ∼10^−4^ W cm^−2^ range. The photocurrent was measured though a Lakeshore preamplifier connected to a Stanford Research Systems lock-in amplifier set to voltage mode. Power dependent responsivity was measured with a QPhotonics 975 nm laser diode controlled by a Newport 560B laser driver and an Agilent Waveform generator. Light intensity was calibrated with an Ophir PD300-IR germanium photodiode. The time response of the devices was characterized with the same setup at different modulation frequencies. Time traces were acquired with an Agilent Infiniium Oscilloscope (DSO8104A) across a 50 Ω input impedance triggered by the laser diode driving pulse.

Noise characterization: noise measurements were carried out using a Lakeshore preamplifier connected with the Stanford Research System SR830 at different bias and frequency conditions. In-phase and quadrature components were monitored until they reached a steady state value within a 20% interval during at least 30 min. The performance of the best optimized devices was confirmed in at least in three independent experiments (Supplementary Fig. 18).

### *In operando* transient absorption spectroscopy

A regeneratively amplified Yb:KGW laser (Light Conversion Pharos) was used to generate femtosecond pulses (1,030 nm, 5 kHz). A portion of this pulse was passed through an optical parametric amplifier (Light Conversion Orpheus), generating pump pulses of either 450 nm (to observe the bleach signal in the quantum dots) or 1,000 nm light (to observe the transfer from the quantum dots into the perovskite). The pump and remainder of the fundamental were sent into an optical bench (Ultrafast Helios), where the frequency of the pump was halved to 2.5 kHz with an optical chopper. The time delay between pulses was controlled by sending the fundamental through a delay stage; after which, the fundamental was focused onto a near-infrared continuum generation crystal (Ultrafast), producing a white-light continuum probe pulse in both the visible and near-infrared regions. The experiments were carried out in reflection mode, where the probe was reflected from the gold electrode of the full device and directed toward the detector (Ultrafast Helios). External bias was provided with a Keithley 2400 source metre. The sample was translated at 1 mm s^−1^, and, depending on the strength of the signal, a number of bidirectional scans were averaged to assist with lowering the noise. After the sample, colour filters were selected to transmit either the visible or near-infrared portion of the probe. The power of the pump pulse was set to 11 μJ cm^−2^ for 450 nm and 225 μJ cm^−2^ for 1,000 nm. The larger fluence for the near-infrared pump was used due to smaller signal from the quantum dots. Numerical fittings were performed to the decay traces after subtracting negative time signal background.

### Numerical modelling

CQD photodetector devices were modelled with SCAPS simulation software. Simulation details are available in Supplementary Fig. 1 and Supplementary Table 1. Field lines in Fig. 2 have been simulated with COMSOL Multiphysics Electrostatic solver based on the electronic properties of CQD and perovskite materials. Details on the calculation of capture and emission rates can be found in Supplementary Note 1.

### Data availability

All data are available upon request.

## Additional information

**How to cite this article:** García de Arquer, F. P. *et al*. Field-emission from quantum-dot-in-perovskite solids. *Nat. Commun.* **8,** 14757 doi: 10.1038/ncomms14757 (2017).

**Publisher's note**: Springer Nature remains neutral with regard to jurisdictional claims in published maps and institutional affiliations.

## Supplementary Material

Supplementary Information Supplementary Figures, Supplementary Tables, Supplementary Notes and Supplementary References
