# Supplementary material for: Field-emission from quantum-dot-in-perovskite solids
Source: Nat Commun. 2017 Mar 24;8:14757. doi: 10.1038/ncomms14757 (PMC5376666; doi:10.1038/ncomms14757)
Supplement: Supplementary Information — Supplementary Figures, Supplementary Tables, Supplementary Notes and Supplementary References [file ncomms14757-s1.pdf]

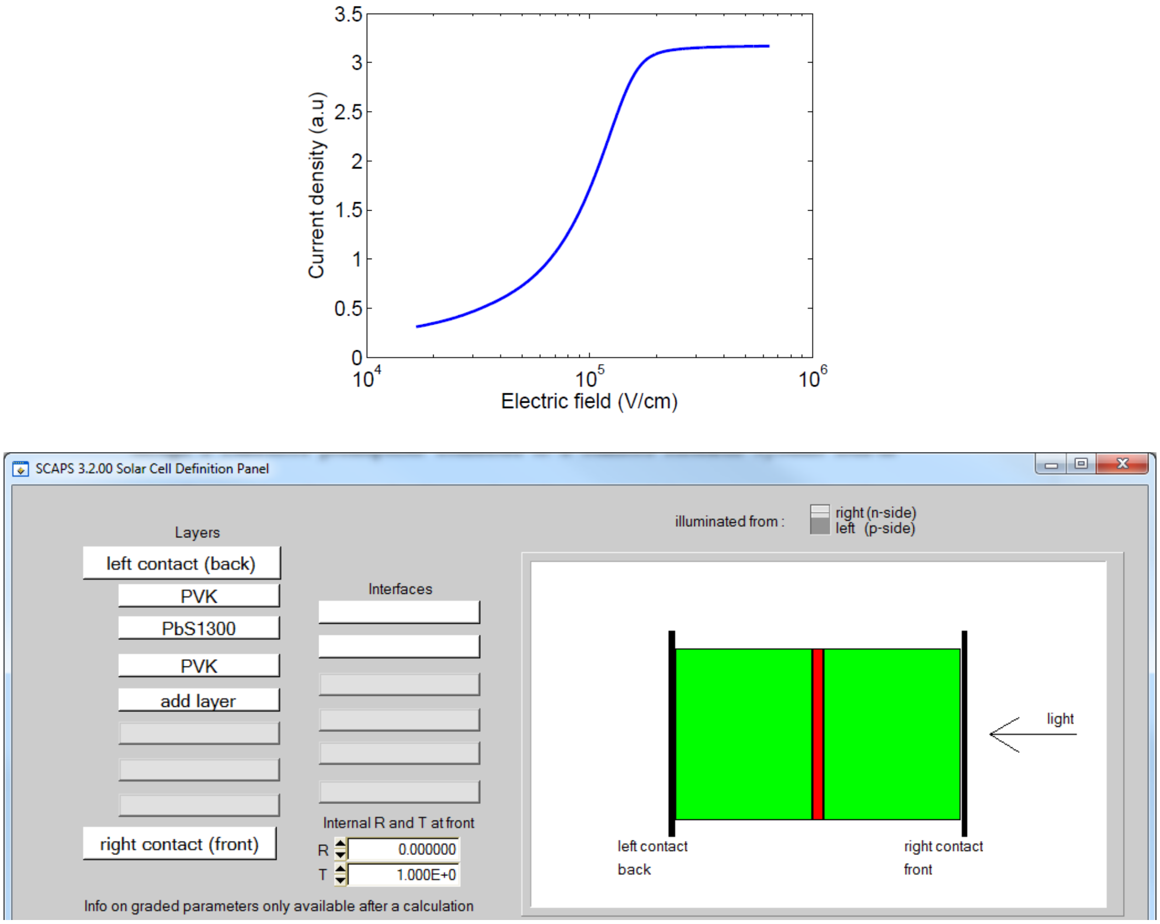

Supplementary Figure 1 | SCAPS simulation and parameter space: (top) Current-density as a function of applied electric field for a perovskite-CQD-perovskite stack. (bottom) Screenshot of the 1D structure used to simulate transport across a perovskite/CQD/perovskite junction. The simulation parameters are listed in Supplementary Table 1.

Supplementary Table 1| Details of SCAPS simulations and used parameters.

	Perovskite layer	CQD layer
Thickness (nm)	75 nm	6 nm
Bandgap edge (eV)	1.53	0.9
Electron affinity (eV)	3.9	4.1
Permittivity (ε_r)	60	22
CB/VB DOS (cm^{-3})	10^{19}	10^{19}
Electron mobility (cm^2/Vs)	6	$2\cdot10^{-2}$
Hole mobility (cm^2/Vs)	19	$2\cdot10^{-2}$
N_{donor} (cm^{-3})	10^{15}	10^{14}
$N_{acceptor}$ (cm^{-3})	10^{15}	10^{16}

Supplementary Note 1.

Influence of host mobility in the carrier capture probability

The carrier trap probability of a carrier traversing a CQD embedded in the QDiP solid was calculated as follows:

$$\eta_{trap} = \frac{\tau_{th}}{\tau_{tra} + \tau_{th}} \qquad \text{(eq. 1)}$$

where τ_{tra} is the transverse time though the dot conduction/valence band, which we estimated as τ_{tra}.= d_{dot}/v_{tra}, where $v_{tran} = \mu E$ is the mobility-field product. d_{dot}~5.5 nm represents the dot diameter. It is important to note that due to the electronic delocalization that arises as a consequence of the epitaxial incorporation of the quantum dots, the mobility is not that of the CQDs but that of the QDiP solid.[1] The thermalization lifetime τ_{th}.is varied. An electric field of 10^6 V·cm^{-1} is assumed for the calculations.

Carrier capture rate as a function of applied electric field.

The carrier capture probability ($\eta_{capture}$) of carriers in the QDiP phase is calculated as follows. First, the probability for a carrier to find a quantum dot:

$$\eta_{CQD} = \sigma_{CQD} \cdot \langle v \rangle \cdot N_{host} \qquad \text{(eq. 2)}$$

where $v = v_{th} + v_{drift}$ comprises both thermal and drift components. σ_{CQD} is the cross-Section of the CQDs. N_{host} is the carrier concentration of the host medium, in this case the $MAPbI_{2.5}Br_{0.5}$ perovskite. The capture probability can be obtained then as,

$$\eta_{capture} = \eta_{CQD} \cdot \eta_{trap} \qquad \text{(eq. 3)}$$

A flat, field-independent component appears as a consequence of the thermal motion of the carriers in the QDiP solid. This increases substantially once the drift velocity surpasses the random, thermal motion, and finally saturates once carriers traverse the dot before thermalization occurs.

Thermionic emission of carriers

Once carriers are photogenerated or trapped into the CQDs they can escape if thermally emitted over the potential barrier. The rate of emission can be calculated as follows:

$$GT_j = \sigma_{CQDs} \cdot v_{th} \cdot N_{DOS} \cdot \exp(-E_{bj}/kT) \qquad \text{(eq. 4)}$$

where j represents the type of carrier (j=e for electrons or j=h for holes). N_{DOS} stands for the density of available states in the host material conduction or valence band. E_{bj} is the activation barrier for the j-type of carrier.

Fowler-Nordheim carrier emission

If the electric field is high enough, carriers will start tunneling through the triangular potential barrier established between the host and the sensitizer. This field-emission process can significantly boost the injection rate by orders of magnitude.[2]

$$GFN_j = \frac{E}{4\sqrt{2qm \cdot E_{bj}}} \cdot \exp\left(\frac{-4\sqrt{2qm} \cdot E_{bj}^{3/2}}{3q\hbar E}\right) \qquad \text{(eq. 5)}$$

Supplementary Note 2.

Emission of QDs photocharges into the perovskite

We carried out transient absorption spectroscopy measurements to characterize the ultra-fast field emission dynamics of photocharges from the QDs into the perovskite matrix. When we pump both perovskite and QDs phases, we observe that a faster bleaching occurs at the exciton peak when a reverse electric field of 2×10^5 V·cm^{-1} is applied (main manuscript Fig. 2). This has a two-fold implication:

(1) That photocharges are being emitted out of the QDs

(2) That, under high enough electric fields, photocharges within the perovskite are not being majoritarily captured into the QDs; or, in case they did, emission from the QDs outcompetes this process.

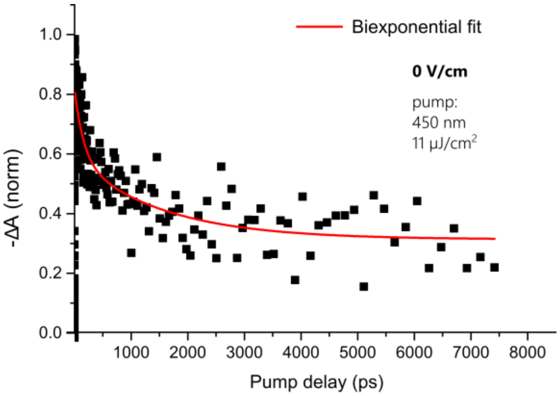

Model	ExpDecay2		
Equation	y = y0 + A1*exp(-(x-x0)/t1) + A2*exp(-(x-x0)/t2)		
Reduced Chi-Sqr	0.00648		
Adj. R-Square	0.8164		
		Value	Standard Error
D	y0	0.31337	0.02637
D	x0	-25.99258	--
D	A1	0.27471	--
D	t1	1555.68986	692.4451
D	A2	0.27266	--
D	t2	163.83188	65.40069

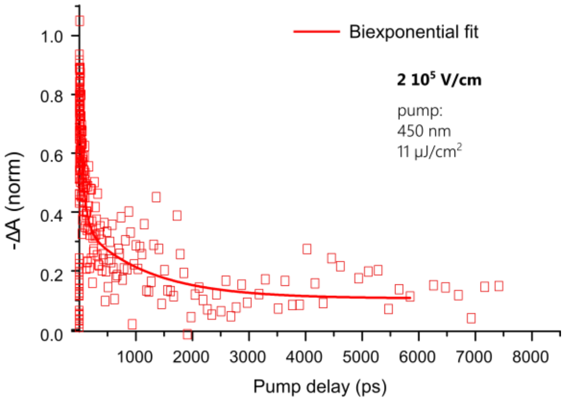

Model	ExpDecay2		
Equation	y = y0 + A1*exp(-(x-x0)/t1) + A2*exp(-(x-x0)/t2)		
Reduced Chi-Sqr	0.01042		
Adj. R-Square	0.82288		
		Value	Standard Error
B	y0	0.10588	0.02989
B	x0	-6.64553	--
B	A1	0.46149	--
B	t1	75.92568	17.57122
B	A2	0.25215	--
B	t2	1172.95222	552.37772

Supplementary Figure 2 | Emission of QD photocharges in quantum-dot-in-perovskite photodiodes and associated dynamics for (left) unbiased devices and (right) biased devices.

Collection of QDs photocharges

We then monitored the visible absorption spectrum of the QDiP photodiode. We observed that the bleach corresponding to the perovskite phase is slowed down (i.e., it is receiving carriers) under the application of a reverse electric field of 2×10^5 $V\cdot cm^{-1}$ (Supplementary Figure 3).

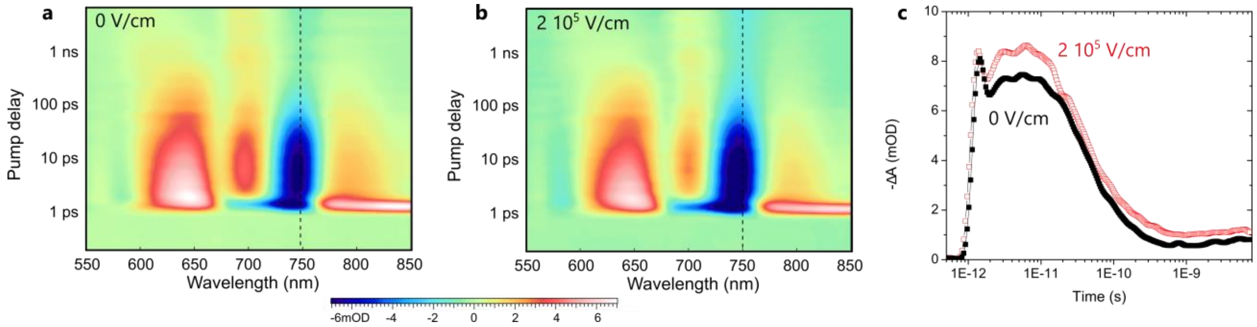

Supplementary Figure 3 | Collection of QD photocharges emitted into the perovskite phase. (a) Transient absorption spectra at 0 V·cm^{-1} and (b) 2×10^{5} V·cm^{-1}. (c) Time evolution slices at 750 nm. The data was fitted with a single exponential decay (Supplementary Table 2). A 1000 nm pump at a fluence of 225 μW·cm^{-2} was used in the experiment to excite the quantum dots.

Supplementary Table 2 | Fitting parameters of the bleach kinetics

	τ_1
**0 V·cm^{-1}**	59±0.8 ps
**2×10^{5} V·cm^{-1}**	52±0.9 ps

Kinetics of QDs photocharges

As a control reference we monitored the transient absorption of CQD only devices, and we observe no significant difference in the bleaching kinetics of this sample (Supplementary Figure 5).

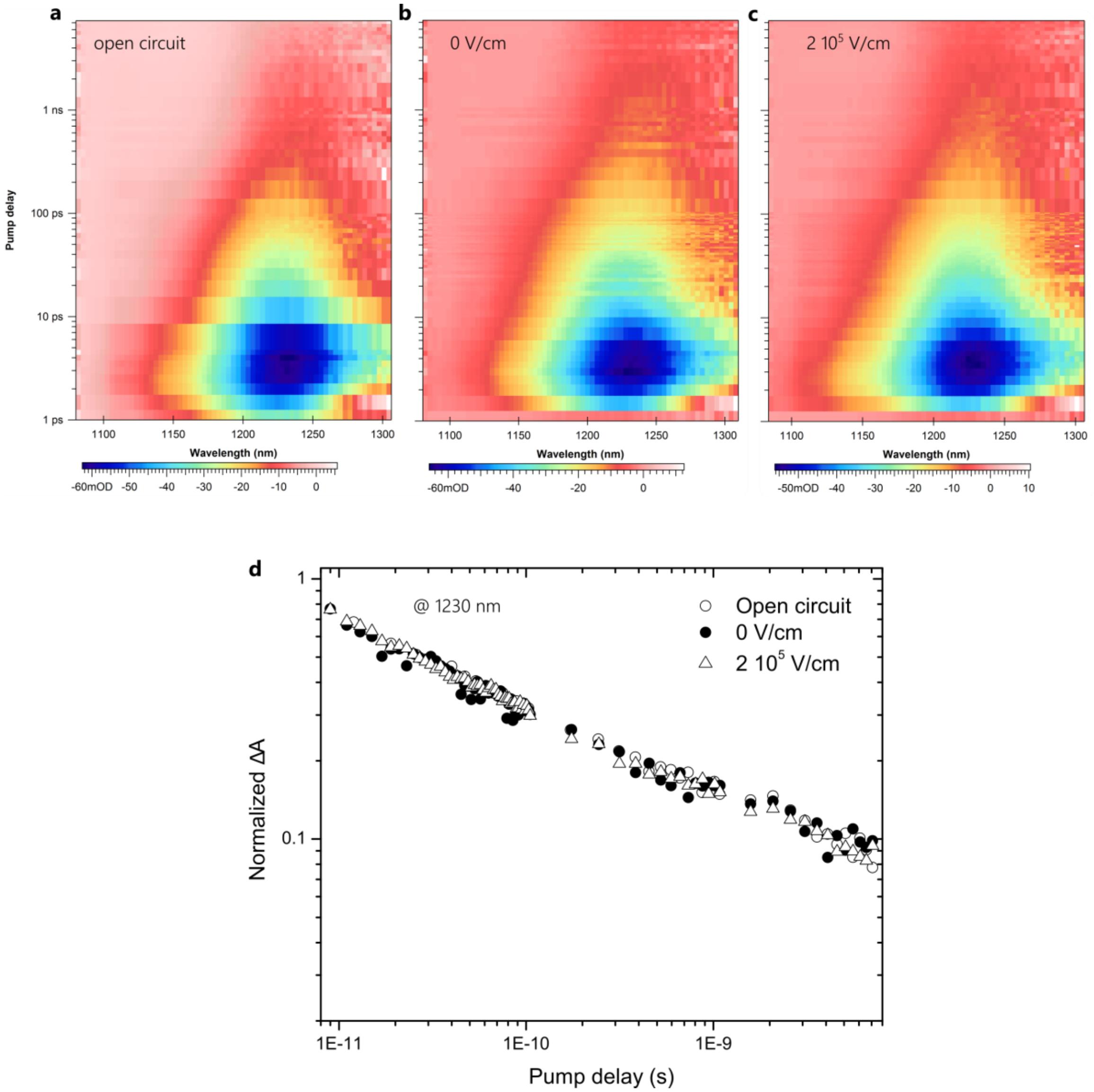

Supplementary Figure 4 | Transient absorption at the QD exciton peak under different electric fields. (a-c). Absorption maps at open circuit, 0 V·cm-1 and 2×10^5

V·cm^{-1} (d) No significant different in the decay is observed at the different scenarios. Pump: 450 nm at a fluence of 11 µJ·cm^{-2}.

Transient absorption of perovskite photodiodes

As a control reference we monitored the transient absorption of perovskite only devices, and we observe no significant difference in the bleaching kinetics of this sample (Supplementary Figure 6).

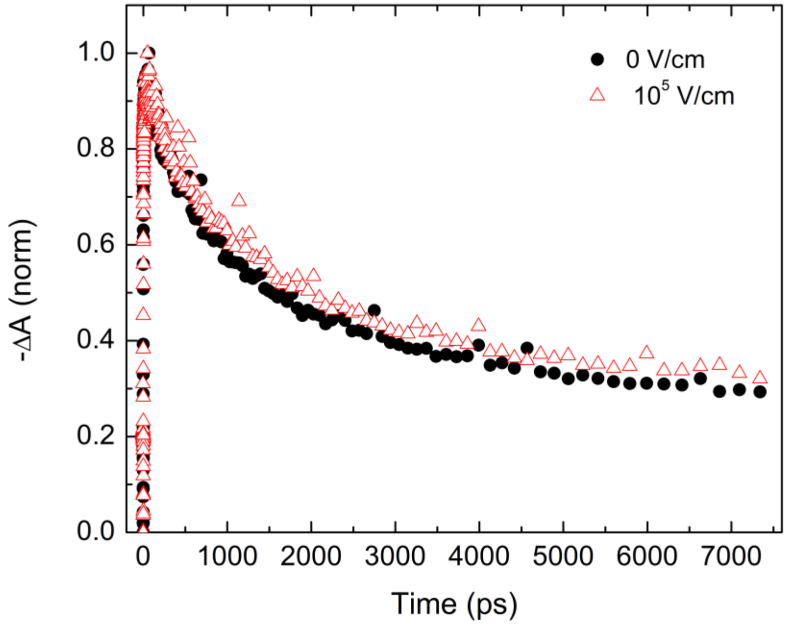

Supplementary Figure 5 | Transient absorption of perovskite diodes under different electric fields. No significant different in the decay is observed at the different scenarios. Pump: 450 nm at a fluence of 11 µJ·cm^{-2}.

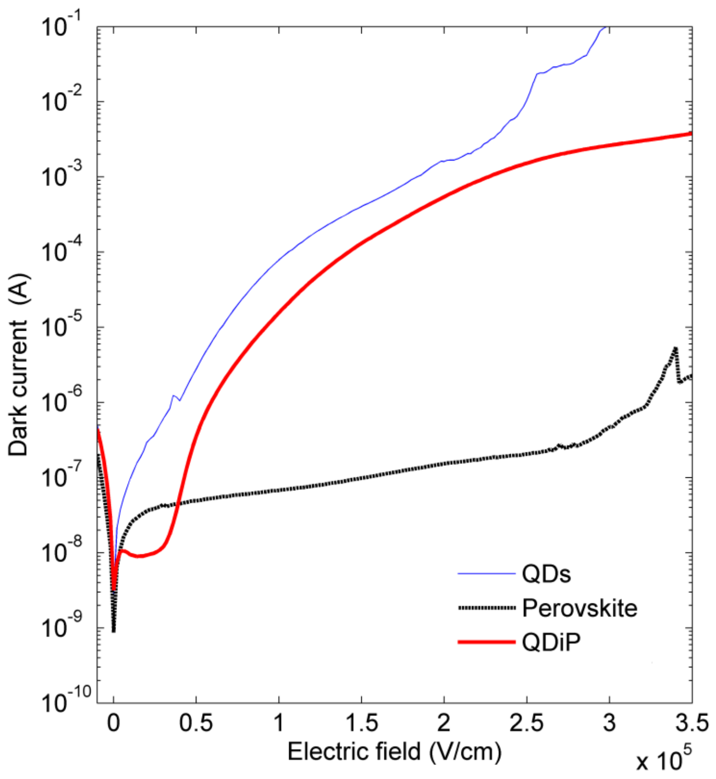

Supplementary Figure 6 | Dark current-voltage characteristics: Reverse current-voltage characteristics for QDiP (red) and perovskite photodiodes (black). Above $3.5{\times}10^5$ V·cm^{-1} the perovskite sample breaks down with irreversible damage. The *J-V* curve for QDs only device using the same architecture is shown for comparison.

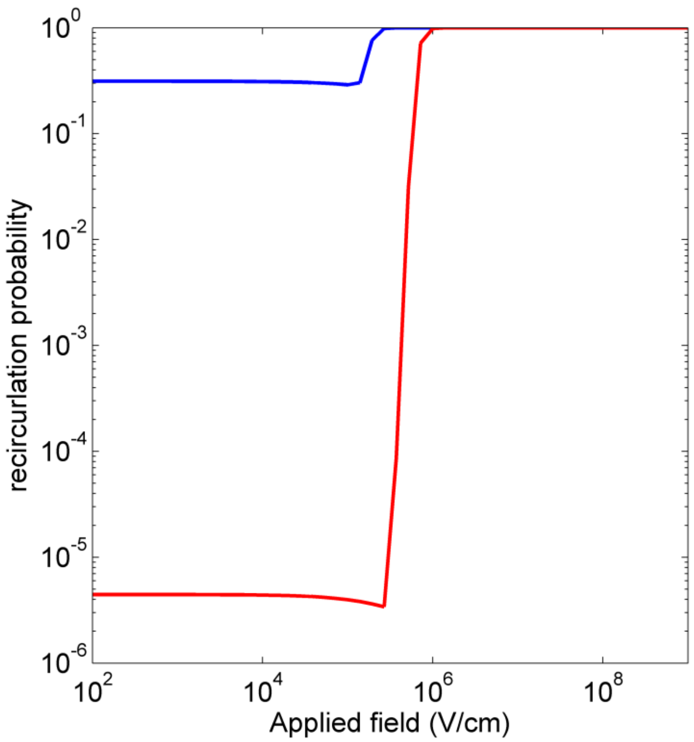

Supplementary Figure 7 | Recirculation probability. Predicted recirculation probability of electrons (blue) and holes (red) as a function of applied field. As a result of the recirculation imbalance of different charge types photomultiplicative gain effects appear.

Supplementary Note 3.

Estimation of the internal gain

The internal gain (or internal quantum efficiency, IQE) was estimated by dividing the EQE by the double pass absorption (A) of the measured device.

$$IQE(\lambda,V) = EQE(\lambda,V)/A(\lambda) \qquad \text{(eq. 6)}$$

At 1V reverse bias we obtain an EQE of 20.34% at 975 nm (Figure 3c in the main text). The absorption at this wavelength is shown in Figure S5, as measured with a Perkin Elmer UV-VIS-IR spectrometer equipped with an integrating sphere (see methods). The resulting (estimated) IQE or internal gain is approximately unity at this condition.

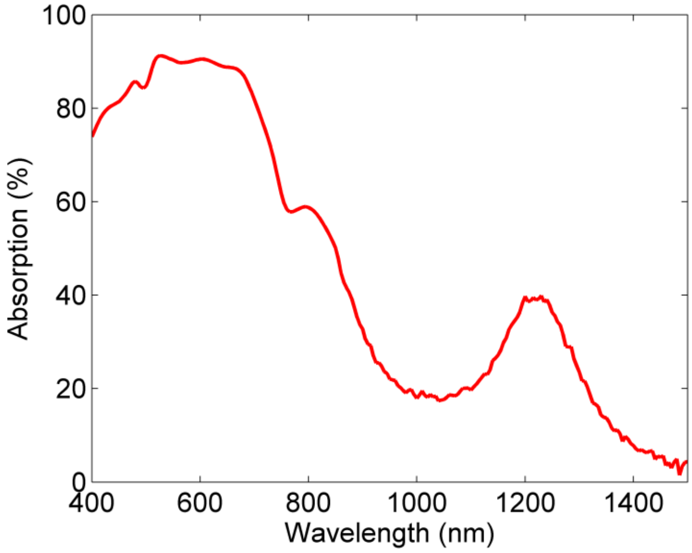

Supplementary Figure 8 | Double pass absorption A(%) = 100 – R(%) – T(%) of a typical DiP device. At 975 nm an absorption value of 19.6% is measured. This is consistent with the double pass absorption expected for a 1:1 Perovskite:CQD concentration with an absorption coefficient of 10^4 cm^{-1} and 200 nm thick film.

Supplementary Note 4.

Bias-dependence of carrier emission

The contribution of the quantum-dots in the SWIR region of the EQE increases with bias. This is attributed to two different mechanisms:

- A reduced capture probability as the reverse bias increases. This increase is spectral independent and saturates after -1V (in agreement with our model, which predicts this voltage to be the threshold where Fowler-Nordheim tunnelling drives the emission of photocharges from the quantum-dot into the perovskite). This is especially evident at visible wavelengths, where the perovskite is also contributing to photocurrent (Fig. 3b in the main manuscript).
- The increasing probability for the emission of charges from the quantum dots into the perovskite as the reverse bias increases (Supplementary Figure 8). This yields up to a 60-fold increase in the EQE at the short-wavelength infrared, where the quantum dots are the only absorbing phase. The increasing contribution of the quantum-dots in the SWIR with bias is more significant as their concentration with respect to the perovskite host-phase is reduced (Supplementary Figure 9). An increase of over two orders of magnitude is recorded in this case.

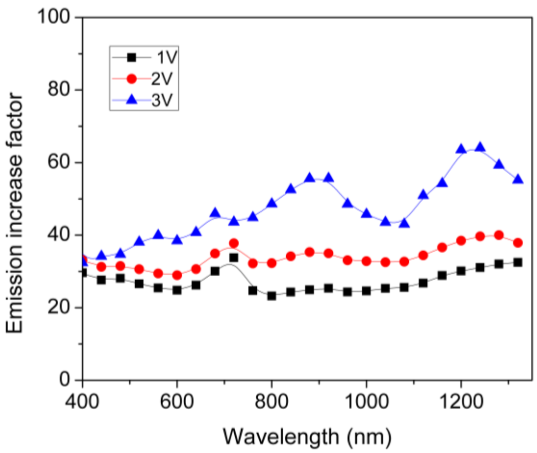

Supplementary Figure 9 | Spectral dependence of the increase in emission under different reverse biases (1:1 quantum dot:perovskite ratio). We define the emission increase as the ratio between the EQE at a bias Vj and the EQE at short-circuit conditions. At the infrared region, photocharge emission experiences a higher growth as the yield of carriers that are injected into the $MAPbI_{2.5}Br_{0.5}$ matrix increases.

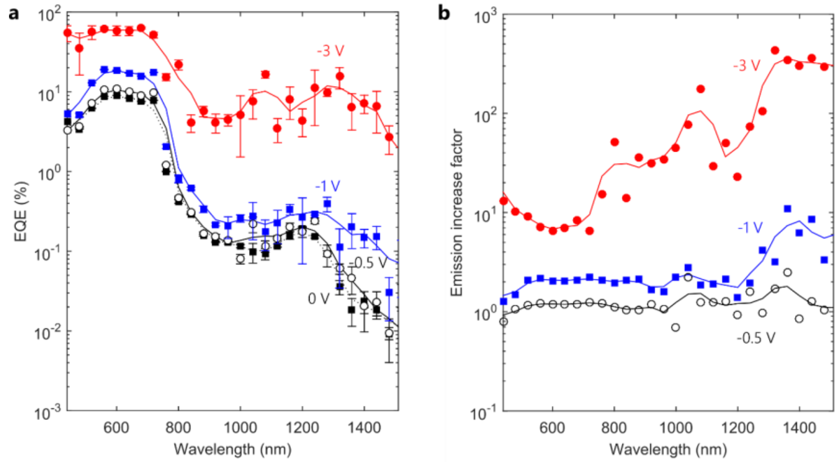

Supplementary Figure 10 | Spectral dependence of the increase in emission under different reverse biases (1:10 quantum dot:perovskite ratio). The emission increase factor is calculated as the ratio of the external quantum efficiency

at a given bias compared to that obtained at 0V. An increase of over two orders of magnitude is recorded in the infrared region of the spectrum.

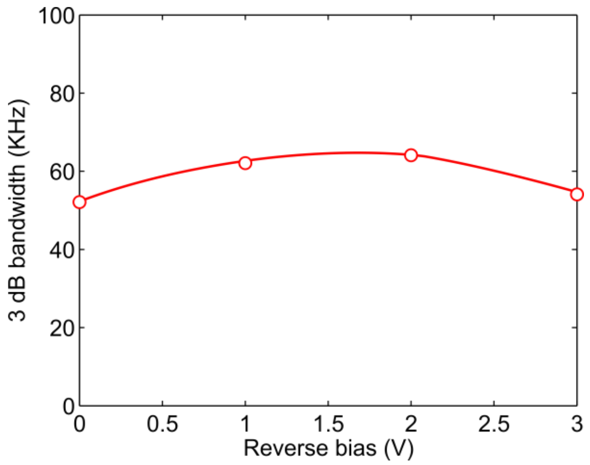

Supplementary Figure 11. | Temporal response as a function of reverse bias. 3dB bandwidth as a function of reverse bias under 100 μW·cm^{-2} 975 nm illumination.

Supplementary Note 5.

Noise reduction with temperature

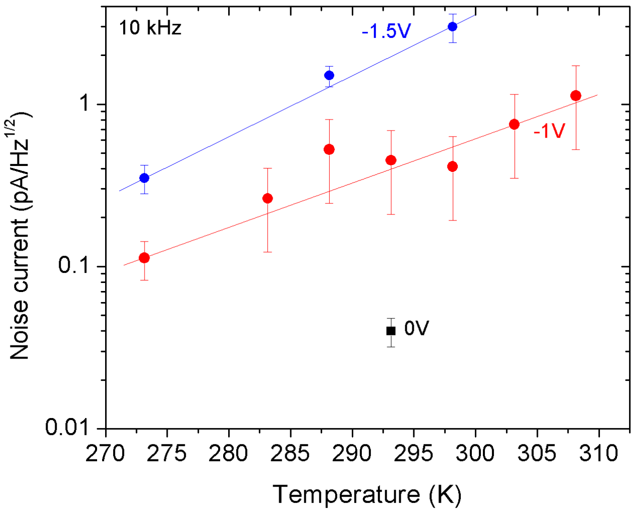

Supplementary Figure 12 | Noise current as a function of temperature. The noise current was measured at various operating temperatures. Device temperature was controlled and monitored with a Laird Thermoelectric Temperature Controller. A 30 min stabilization window was used prior to noise measurement for each temperature. Noise traces of 30 min were monitored with a Stanford SR-830 lock-in amplifier as described in the experimental Section. Error bars correspond to the standard deviation over at least 20 consecutive measurement

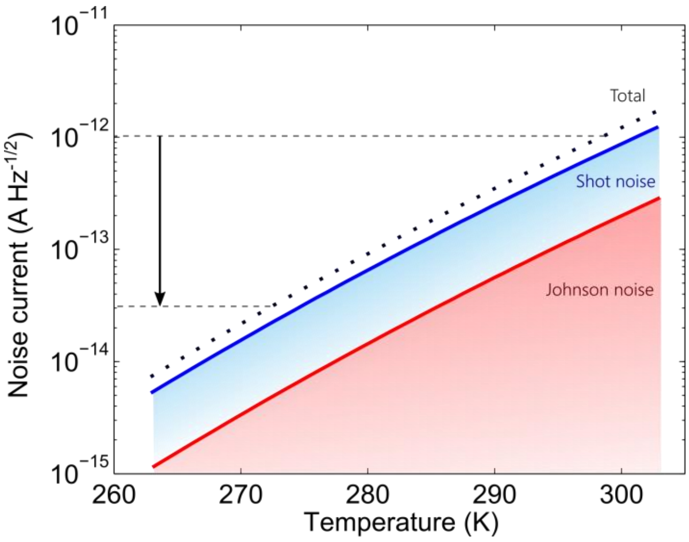

Supplementary Figure 13 | Noise reduction with temperature. The decrease in the operation temperature of the device results in a reduction of the dark current (I_d) and associated to this, the noise current (I_N).

The potential decrease in I_N was calculated from the Shot and Johnson components, which are the major contribution to the measured noise as it is shown in Supplementary Figure 12. These were calculated as,

$$I_{shot} = (2qI_d)^{1/2} \quad \text{(eq. 7)}$$

$$I_{Johnson} = (4kT/R)^{1/2} = (4k\,T\,I_d/V)^{1/2} \quad \text{(eq. 8)}$$

where q is the elementary charge (1.6021×10^{-19} C), k the Boltzmann constant (1.38×10^{-23} m^2 Kg s^{-2} K^{-1}), T the operation temperature and V the applied bias. The dependence of I_d with temperature was calculated based on Sze and modelled as,

$$I_d = BT^3\exp(-E_{g0}/kT) \quad \text{(eq. 9)}$$

where B is a constant independent of T, and E_{g0} is bandgap of the $MAPbI_{2.5}Br_{0.5}$ perovskite. A reduction in the operation temperature of 25K comes associated with a projected 30-fold reduction in the noise, which supports the experimentally measured 10-fold reduction.

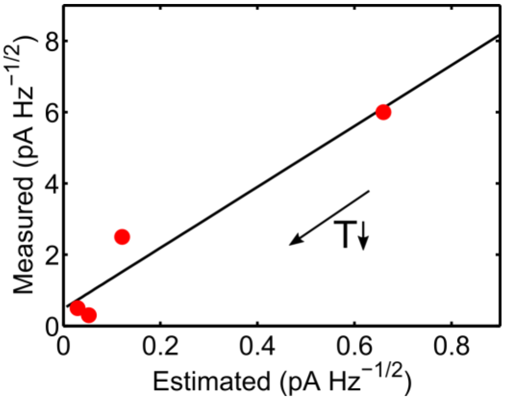

Supplementary Figure 14 | Correlation of measured and shot-estimated noise.

Supplementary Table 3 | Photodetector benchmark. NEP measured in (pW $Hz^{-1/2}$). A_d represents the electrical active area of the photodetector.

	NEP (reported)	NEP (measured)	A_d (cm^2)	D* (Jones)
Ge (818IR)	0.7	0.6	0.071	4.44×10^{11}
InGaAs (918IG)	0.03	0.04	0.071	6.66×10^{12}
**Field-Emission QDiP**		**0.06***	**0.07**	**3.92×10^{12}**

*at the exciton peak (~1240 nm)

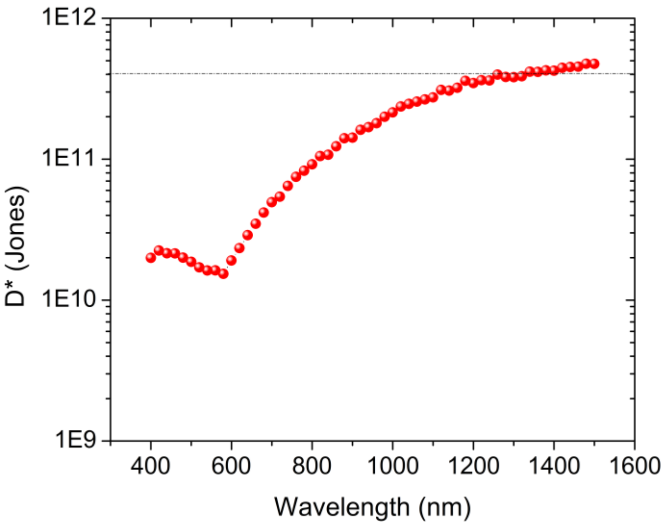

Supplementary Figure 15 | Normalized detectivity spectra of the 818-IR Ge photodetector. The dashed line depicts the value reported by the manufacturer (Newport)

Supplementary Note 6.

Continuous operation

We monitored the photoresponse of a typical device over the course of an initial 100 h study. The device was subjected to continuous illumination of 10 kHz pulsed 1mWcm^{-2} 974 nm laser light and biased at -1V reverse voltage. Prior to operation, the device was encapsulated in a nitrogen-filled glovebox with a UV-curable epoxy. The photoresponse was monitored with an oscilloscope at different intervals.

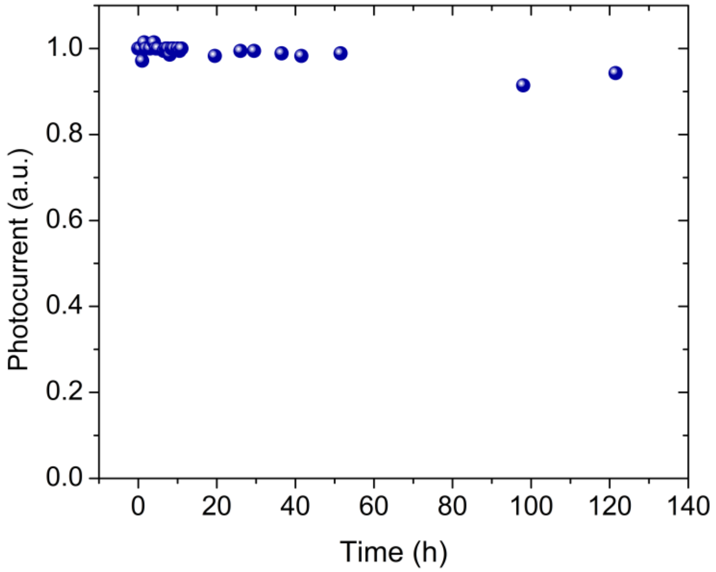

Supplementary Figure 16 | Continuous device operation at -1V applied bias.

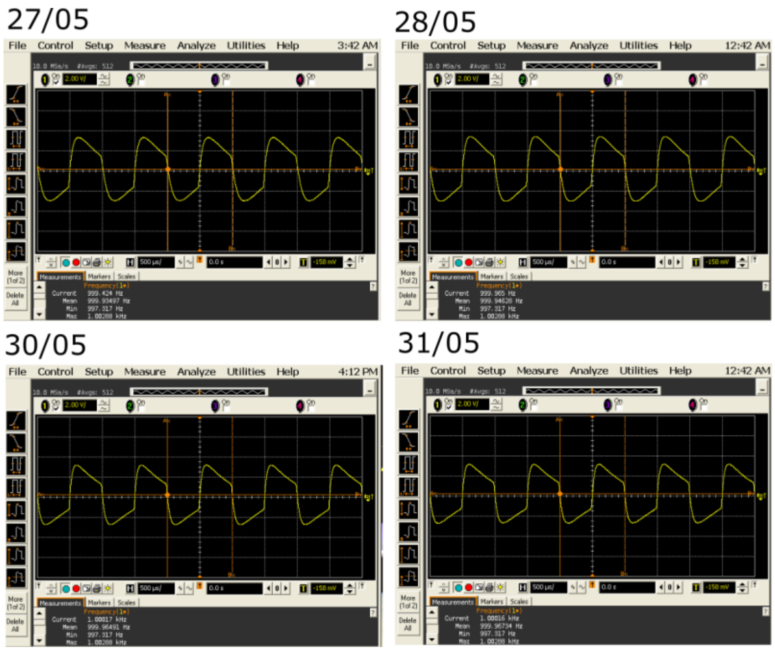

Supplementary Figure 17 | Photoresponse traces along the stability study.

Supplementary Note 7.

RoHs of lead in optoelectronic devices

Pb is a regulated material in certain jurisdictions such as Europe. RoHS exemption 7c allows use of lead in electronic ceramic parts. Additionally, RoHS exemption 7a allows for Pb in products as long as it is below 0.1% by mass. Here we show that, integrated on a typical substrate for sensing or imaging, Pb is in fact in the 100 ppm range (well below the 1000 ppm limited permitted by RoHs).

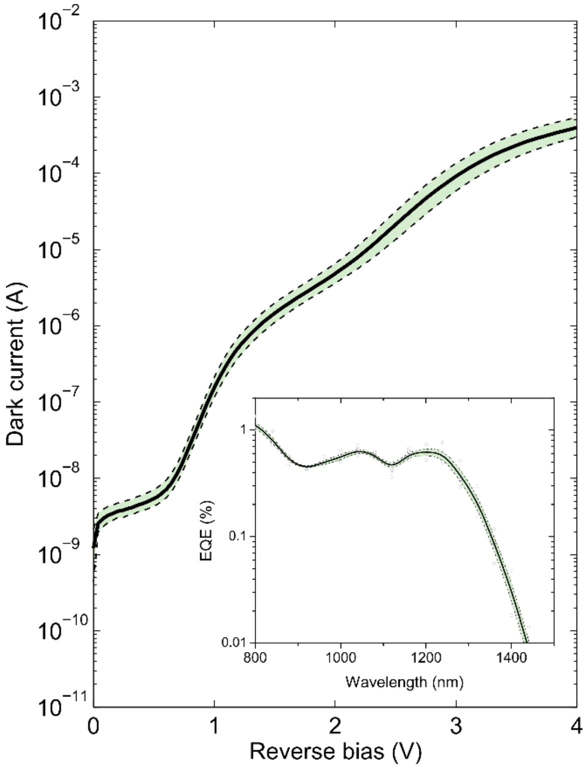

Supplementary Figure 18 | Device reproducibility. Dark current traces and external quantum efficiency spectra taken over different independent devices. In the IV plot we represent the average of the samples with an 80% confidence interval as calculated from the standard deviation of 5 different samples. A similar plot is displayed in the inset for the external quantum efficiency at short circuit conditions.

Supplementary references

1. Ning, Z. *et al.* Quantum-dot-in-perovskite solids. *Nature* **523,** 324–328 (2015).
2. Sze, S. M. *Physics of Semiconductor Devices*. (John Wiley & Sons, 1981).
